# Supplementary material for: Genomic perspectives on foodborne illness
Source: medRxiv. 2024 May 16:2024.05.16.24307425. Preprint. [Version 1] doi: 10.1101/2024.05.16.24307425 (PMC11188124; doi:10.1101/2024.05.16.24307425)
Supplement: Supplement 1 [file media-1.pdf]

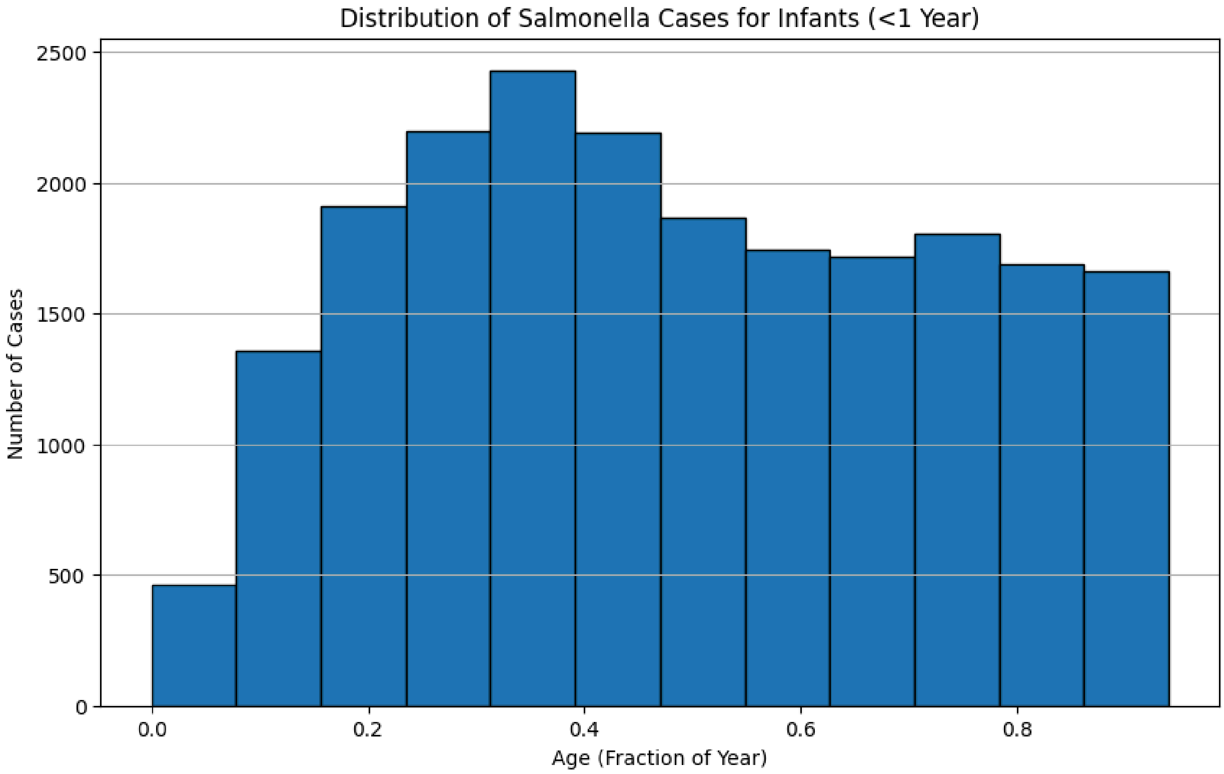

Supplementary Figure 1

Number of Salmonella cases for infants < 1 year of age in fractions of one year.
